# Supplementary figures and images for: Risk of fatty liver after long-term use of tamoxifen in patients with breast cancer
Source: PLoS One. 2020 Jul 30;15(7):e0236506. doi: 10.1371/journal.pone.0236506 (PMC7392315; doi:10.1371/journal.pone.0236506)

**
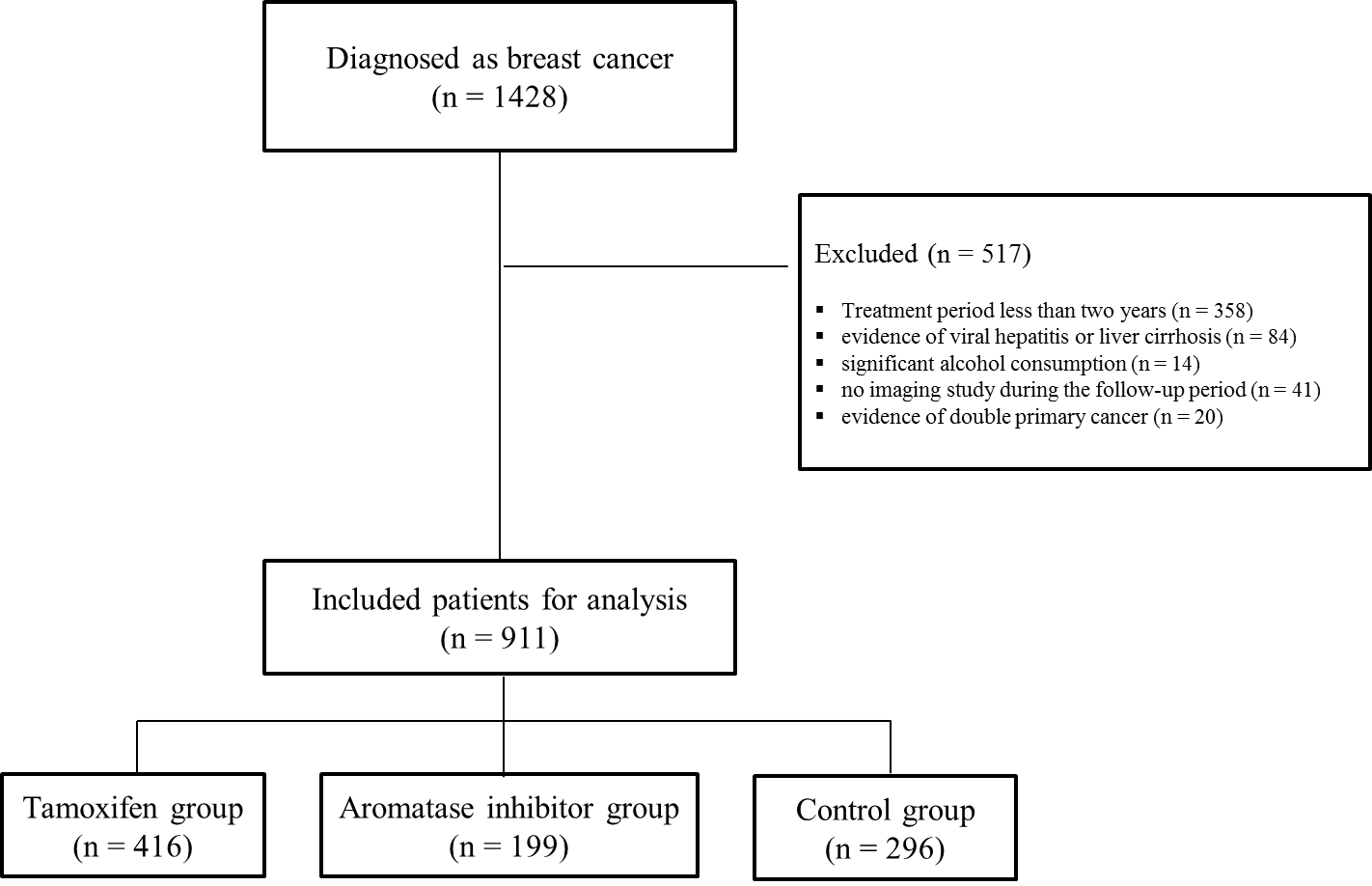
**

**Supplementary Figure 1.** The flow chart of patients assessed for eligibility

Supplement: S1 Fig — (DOCX) [file pone.0236506.s001.docx]
