# Supplementary material for: Risk of fatty liver after long-term use of tamoxifen in patients with breast cancer
Source: PLoS One. 2020 Jul 30;15(7):e0236506. doi: 10.1371/journal.pone.0236506 (PMC7392315; doi:10.1371/journal.pone.0236506)
Supplement: S2 Fig — (DOCX) [file pone.0236506.s002.docx]

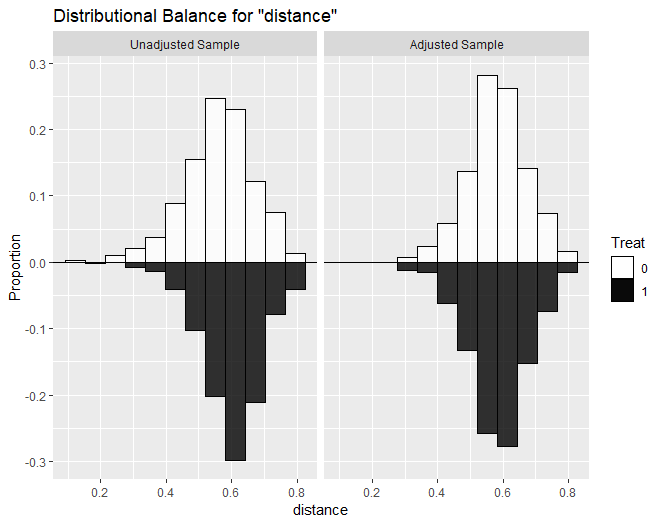


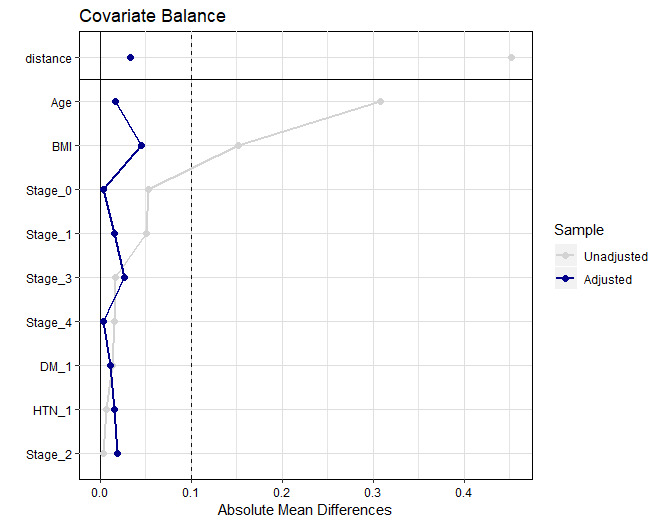


**Supplementary Figure 2.** Distribution of propensity score for balance between groups and covariance balance plot
