# Supplementary material for: Risk of fatty liver after long-term use of tamoxifen in patients with breast cancer
Source: PLoS One. 2020 Jul 30;15(7):e0236506. doi: 10.1371/journal.pone.0236506 (PMC7392315; doi:10.1371/journal.pone.0236506)
Supplement: S3 Fig — (A) Before matching, (B) After matching. (DOCX) [file pone.0236506.s003.docx]

**
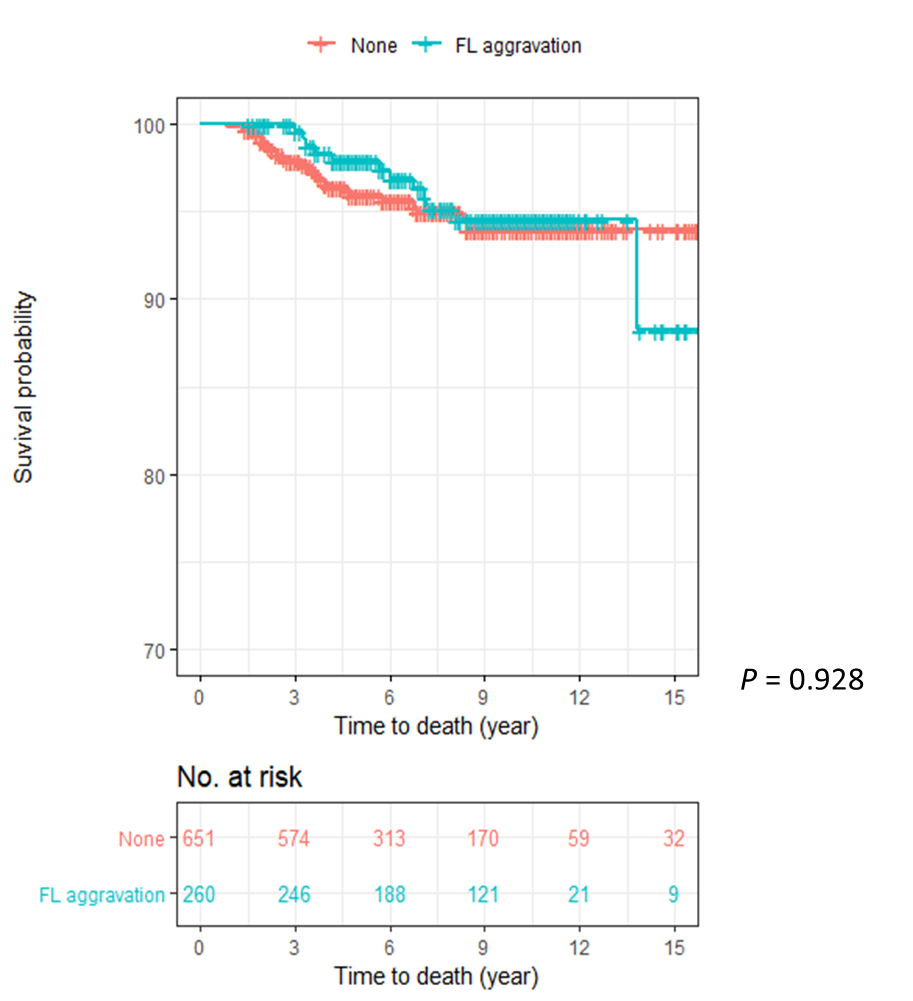
**(A)


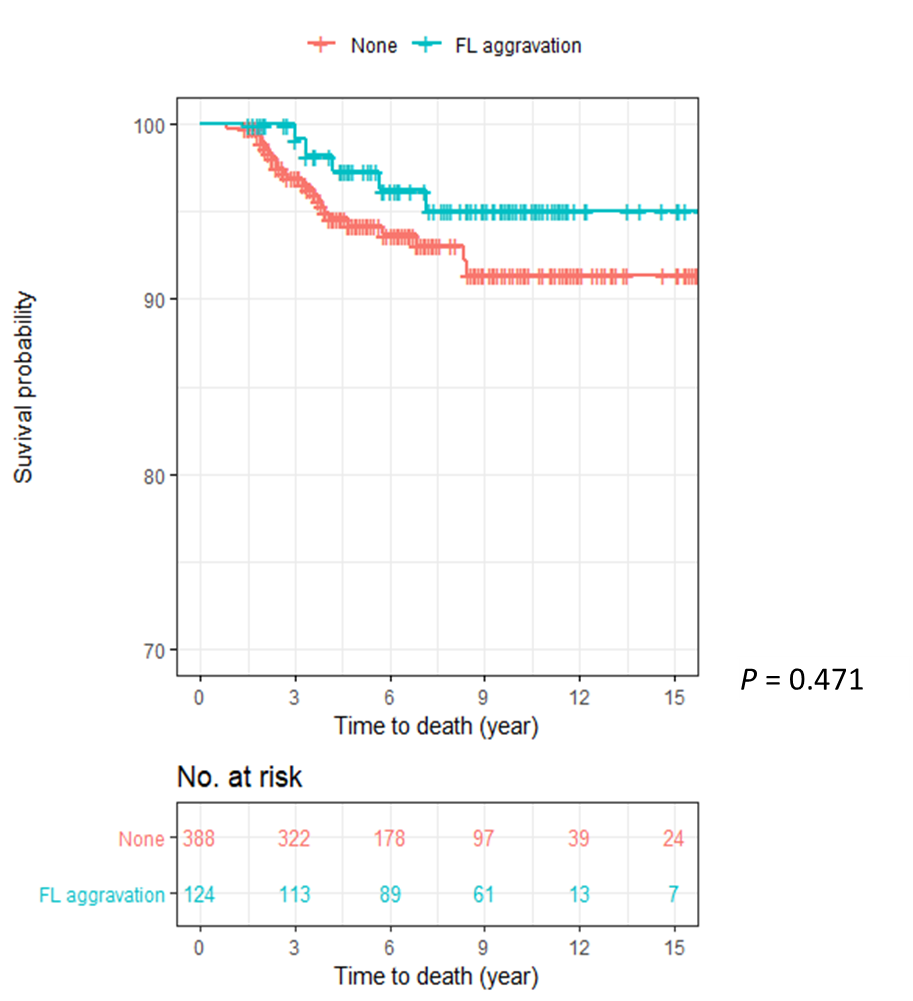
(B)

**Supplementary Figure 3.** Difference in survival rate according to aggravation of fatty liver.

(A) Before matching, (B) After matching.
