# Supplementary material for: Risk of fatty liver after long-term use of tamoxifen in patients with breast cancer
Source: PLoS One. 2020 Jul 30;15(7):e0236506. doi: 10.1371/journal.pone.0236506 (PMC7392315; doi:10.1371/journal.pone.0236506)
Supplement: S1 Table — (DOCX) [file pone.0236506.s004.docx]

**Supplementary Table 1. Multivariable Cox proportional hazards regression for fatty liver aggravation (Before matching)**

| **Variable** | **Multivariable** | |
| --- | --- | --- |
|  | **HR (95% CI)** | **p-value** |
| **All (N=911)** |  |  |
| Treatment modality |  |  |
| Control | 1 (Reference) |  |
| Aromatase inhibitor | 1.230 (0.839-1.803) | 0.289 |
| Tamoxifen | 1.598 (1.173-2.177) | 0.003 |
| Body mass index (㎏/㎡) | 1.083 (1.052-1.114) | <0.001 |
| PR (Intermediate or High) | 1.395 (1.049-1.857) | 0.022 |
| FSH | 0.995 (0.991-0.999) | 0.024 |
| Platelet | 1.002 (1.001-1.004) | 0.004 |
| **Fatty liver (-) at baseline (N=651)** |  |  |
| Treatment modality |  |  |
| Control | 1 (Reference) |  |
| Aromatase inhibitor | 1.532 (1.001-2.345) | 0.05 |
| Tamoxifen | 1.519 (1.100-2.098) | 0.011 |
| Body mass index (㎏/㎡) | 1.110 (1.068-1.154) | <0.001 |
| Lymph node metastasis | 1.506 (1.137-1.995) | 0.004 |
| FSH | 0.993 (0.989-0.998) | 0.010 |
| Platelet | 1.002 (1.000-1.004) | 0.014 |
| Total bilirubin | 0.522 (0.292-0.934) | 0.029 |
| **Fatty liver (+) at baseline (N=260)** |  |  |
| Treatment modality |  |  |
| Control | 1 (Reference) |  |
| Aromatase inhibitor | 1.110 (0.581-2.122) | 0.752 |
| Tamoxifen | 2.103 (1.156-3.826) | 0.015 |
| Body mass index (㎏/㎡) | 1.049 (1.005-1.096) | 0.029 |
| PR (Intermediate or High) | 1.559 (0.973-2.5) | 0.065 |
| Radiotherapy | 1.656 (1.152-2.382) | 0.007 |
| AST | 1.007 (0.999-1.015) | 0.096 |

Abbreviations: PR, progesterone receptor; FSH, follicle stimulating hormone ; AST, aspartate aminotransferase;
