# Supplementary material for: Risk of fatty liver after long-term use of tamoxifen in patients with breast cancer
Source: PLoS One. 2020 Jul 30;15(7):e0236506. doi: 10.1371/journal.pone.0236506 (PMC7392315; doi:10.1371/journal.pone.0236506)
Supplement: S2 Table — (DOCX) [file pone.0236506.s005.docx]

**Supplementary Table 2. Propensity score matching analysis**

| **Variable** |  | **After matching** | | | |
| --- | --- | --- | --- | --- | --- |
|  |  | **All** | **Control** | **Tamoxifen** | **p-value** |
|  |  | **(N=512)** | **(N=256)** | **(N=256)** |  |
| Change of fatty liver |  |  |  |  | <0.001 |
| Fatty liver progression (-) |  | 293 (57.23%) | 180 (70.31%) | 113 (44.14%) |  |
| Fatty liver progression (+) |  | 219 (42.77%) | 76 (29.69%) | 143 (55.86%) |  |
| Age (year) |  | 48.19 ± 9.56 | 48.11 ± 9.41 | 48.27 ± 9.72 | 0.388 |
| Body mass index (㎏/㎡) |  | 23.96 ± 3.54 | 24.03 ± 3.63 | 23.88 ± 3.46 | 0.635 |
| Diabetes mellitus |  | 27 (5.27%) | 12 (4.69%) | 15 (5.86%) | 0.692 |
| Hypertension |  | 80 (15.62%) | 38 (14.84%) | 42 (16.41%) | 0.715 |
| Treatment duration (month) |  | 49.48 ± 24.51 | 50.32 ± 30.28 | 48.64 ± 16.90 | 0.218 |
| Cancer-related factor |  |  |  |  |  |
| Stage |  |  |  |  | 0.687 |
| 0 |  | 39 (7.62%) | 19 (7.42%) | 20 (7.81%) |  |
| 1 |  | 224 (43.75%) | 114 (44.53%) | 110 (42.97%) |  |
| 2 |  | 201 (39.26%) | 103 (40.23%) | 98 (38.28%) |  |
| 3 |  | 47 (9.18%) | 20 (7.81%) | 27 (10.55%) |  |
| 4 |  | 1 (0.20%) | 0 (0%) | 1 (0.39%) |  |
| Pathology |  |  |  |  | 0.754 |
| Invasive ductal carcinoma |  | 424 (82.81%) | 213 (83.20%) | 211 (82.42%) |  |
| Ductal carcinoma in situ |  | 46 (8.98%) | 22 (8.59%) | 24 (9.38%) |  |
| Mucinous carcinoma |  | 9 (1.76%) | 3 (1.17%) | 6 (2.34%) |  |
| Infiltrating lobular carcinoma |  | 15 (2.93%) | 7 (2.73%) | 8 (3.12%) |  |
| Intraductal papilloma |  | 4 (0.78%) | 2 (0.78%) | 2 (0.78%) |  |
| Tubular carcinoma |  | 2 (0.39%) | 1 (0.39%) | 1 (0.39%) |  |
| Apocrine carcinoma |  | 0 (0%) | 0 (0%) | 0 (0%) |  |
| Squamous carcinoma |  | 1 (0.20%) | 0 (0%) | 1 (0.39%) |  |
| Medullary carcinoma |  | 5 (0.98%) | 3 (1.17%) | 2 (0.78%) |  |
| Others |  | 6 (1.17%) | 5 (1.95%) | 1 (0.39%) |  |
| Lymph node metastasis |  | 157 (30.66%) | 83 (32.42%) | 74 (28.91%) | 0.443 |
| ER (Intermediate or High) |  | 292 (57.37%) | 98 (38.28%) | 194 (76.68%) | <0.001 |
| PR (Intermediate or High) |  | 277 (54.42%) | 94 (36.72%) | 183 (72.33%) | <0.001 |
| HER2 (Intermediate or High) |  | 170 (33.20%) | 71 (27.73%) | 99 (38.67%) | 0.011 |
| p53 |  | 195 (44.52%) | 104 (49.76%) | 91 (39.74%) | 0.044 |
| Ki67 (≥ 40%) |  | 87 (19.12%) | 66 (29.46%) | 21 (9.09%) | <0.001 |
| Laboratory test |  |  |  |  |  |
| FSH (IU/L) |  | 31.26 ± 29.37 | 29.42 ± 29.84 | 33.72 ± 28.62 | 0.009 |
| Platelet (10^3^ mm^3^) |  | 239.46 ± 71.79 | 248.30 ± 68.27 | 230.62 ± 74.22 | 0.004 |
| AST (U/L) |  | 23.44 ± 12.87 | 21.05 ± 9.46 | 25.84 ± 15.21 | <0.001 |
| ALT (U/L) |  | 21.77 ± 18.08 | 19.34 ± 16.80 | 24.20 ± 19.00 | <0.001 |
| Serum albumin (mg/dL) |  | 4.23 ± 0.45 | 4.37 ± 0.43 | 4.09 ± 0.42 | <0.001 |
| Total bilirubin (mg/dL) |  | 0.59 ± 0.25 | 0.62 ± 0.25 | 0.55 ± 0.24 | <0.001 |
| Total cholesterol (mg/dL) |  | 185.01 ± 35.15 | 186.94 ± 35.40 | 183.08 ± 34.86 | 0.214 |
| Triglyceride (mg/dL) |  | 136.92 ± 104.55 | 148.35 ± 115.83 | 126.97 ± 92.81 | 0.129 |
| HDL-cholesterol (mg/dL) |  | 53.98 ± 13.58 | 52.47 ± 13.06 | 55.57 ± 13.97 | 0.028 |
| LDL-cholesterol (mg/dL) |  | 106.98 ± 30.87 | 108.47 ± 30.88 | 105.35 ± 30.86 | 0.325 |
| Fasting blood glucose (mg/dL) |  | 108.70 ± 27.91 | 106.10 ± 23.68 | 111.30 ± 31.41 | 0.070 |
| BARD |  | 1.95 ± 0.71 | 1.95 ± 0.70 | 1.94 ± 0.71 | 0.819 |
| NFS |  | -1.13 ± 1.32 | -1.30 ± 1.21 | -0.96 ± 1.40 | 0.021 |
| FIB-4 |  | 1.19 ± 0.71 | 1.09 ± 0.59 | 1.29 ± 0.80 | 0.002 |

Abbreviations: ER, estrogen receptor; PR, progesterone receptor; HER2, Human epidermal growth factor receptor 2; FSH, follicle stimulating hormone ; AST, aspartate aminotransferase; ALT, alanine aminotransferase; HDL, high-density lipoprotein; LDL, low-density lipoprotein; NFS, nonalcoholic fatty liver disease fibrosis score; FIB-4, fibrosis-4
