# Supplementary material for: Risk of fatty liver after long-term use of tamoxifen in patients with breast cancer
Source: PLoS One. 2020 Jul 30;15(7):e0236506. doi: 10.1371/journal.pone.0236506 (PMC7392315; doi:10.1371/journal.pone.0236506)
Supplement: S3 Table — (DOCX) [file pone.0236506.s006.docx]

**Supplementary Table 3. Univariable proportional hazards regression for fatty liver progression (After matching)**

| **Variable** | **Total (N=512)** | |  | **Initial fatty liver (-) (N=388)** | |  | **Initial fatty liver (+) (N=124)** | |
| --- | --- | --- | --- | --- | --- | --- | --- | --- |
|  | **HR (95% CI)** | **p-value** |  | **HR (95% CI)** | **p-value** |  | **HR (95% CI)** | **p-value** |
| Treatment modality |  |  |  |  |  |  |  |  |
| Control | 1 (Reference) |  |  | 1 (Reference) |  |  | 1 (Reference) |  |
| Tamoxifen | 1.636 (1.237-2.165) | 0.001 |  | 1.481 (1.075-2.042) | 0.016 |  | 2.241 (1.244-4.038) | 0.007 |
| Age (year) | 1.006 (0.992-1.02) | 0.379 |  | 1.016 (1-1.031) | 0.047 |  | 0.979 (0.948-1.011) | 0.191 |
| BMI (㎏/㎡) | 1.059 (1.022-1.097) | 0.001 |  | 1.080 (1.033-1.129) | 0.001 |  | 1.056 (0.987-1.129) | 0.114 |
| Diabetes | 1.159 (0.648-2.076) | 0.619 |  | 1.156 (0.542-2.468) | 0.707 |  | 1.093 (0.436-2.742) | 0.850 |
| Hypertension | 0.940 (0.652-1.355) | 0.739 |  | 0.847 (0.53-1.353) | 0.487 |  | 1.114 (0.61-2.034) | 0.726 |
| Cancer stage |  |  |  |  |  |  |  |  |
| ≤1 | 1 (Reference) |  |  | 1 (Reference) |  |  | 1 (Reference) |  |
| 2 | 1.235 (0.932-1.635) | 0.141 |  | 1.359 (0.977-1.888) | 0.068 |  | 1.018 (0.539-1.746) | 0.949 |
| ≥3 | 1.124 (0.710-1.779) | 0.617 |  | 1.169 (0.692-1.976) | 0.559 |  | 0.903 (0.348-2.341) | 0.834 |
| Pathology |  |  |  |  |  |  |  |  |
| Invasive ductal carcinoma | 1 (Reference) |  |  | 1 (Reference) |  |  | 1 (Reference) |  |
| Ductal carcinoma in situ | 1.074 (0.683-1.689) | 0.758 |  | 0.908 (0.54-1.529) | 0.717 |  | 1.649 (0.653-4.161) | 0.290 |
| Mucinous carcinoma | 1.209 (0.496-2.946) | 0.675 |  | 0.906 (0.288-2.854) | 0.867 |  | 1.986 (0.48-8.208) | 0.343 |
| Infiltrating lobular carcinoma | 1.300 (0.61-2.771) | 0.496 |  | 1.442 (0.673-3.089) | 0.346 |  | 0 (0-Inf) | 0.997 |
| Intraductal papilloma | 1.805 (0.669-4.874) | 0.244 |  | 1.779 (0.438-7.222) | 0.420 |  | 1.927 (0.467-7.957) | 0.365 |
| Tubular carcinoma | 0.901 (0.126-6.435) | 0.917 |  | 0 (0-Inf) | 0.995 |  | 2.770 (0.378-20.309) | 0.316 |
| Squamous carcinoma | 6.484 (0.9-46.72) | 0.064 |  | 7.002 (0.965-50.775) | 0.054 |  | - |  |
| Medullary carcinoma | 0.913 (0.291-2.863) | 0.876 |  | 0.745 (0.184-3.02) | 0.680 |  | 1.235 (0.17-8.975) | 0.835 |
| Others | 0.826 (0.205-3.333) | 0.788 |  | 0 (0-Inf) | 0.994 |  | 1.504 (0.361-6.27) | 0.575 |
| Lymph node metastasis | 1.056 (0.791-1.41) | 0.711 |  | 1.165 (0.831-1.634) | 0.376 |  | 0.818 (0.468-1.428) | 0.480 |
| ER (Intermediate or High) | 1.699 (1.283-2.252) | <0.001 |  | 1.537 (1.106-2.135) | 0.010 |  | 2.119 (1.229-3.653) | 0.007 |
| PR (Intermediate or High) | 1.753 (1.328-2.313) | <0.001 |  | 1.667 (1.206-2.306) | 0.002 |  | 2.124 (1.232-3.659) | 0.007 |
| HER2 (Intermediate or High) | 1.163 (0.881-1.537) | 0.287 |  | 0.947 (0.679-1.32) | 0.747 |  | 1.903 (1.13-3.203) | 0.015 |
| p53 | 0.76 (0.569-1.017) | 0.065 |  | 0.814 (0.577-1.147) | 0.239 |  | 0.626 (0.362-1.083) | 0.094 |
| Ki67 (≥ 40%) | 0.679 (0.455-1.013) | 0.058 |  | 0.818 (0.523-1.279) | 0.378 |  | 0.399 (0.158-1.003) | 0.051 |
| Chemotherapy | 0.866 (0.618-1.213) | 0.402 |  | 0.889 (0.61-1.296) | 0.541 |  | 0.74 (0.336-1.634) | 0.457 |
| Radiotherapy | 1.117 (0.856-1.459) | 0.414 |  | 0.826 (0.606-1.126) | 0.227 |  | 2.303 (1.366-3.883) | 0.002 |
| Tretment duration (month) | 0.979 (0.972-0.985) | <0.001 |  | 0.977 (0.968-0.985) | <0.001 |  | 0.980 (0.969-0.99) | <0.001 |
| FSH | 0.998 (0.993-1.003) | 0.389 |  | 0.998 (0.992-1.004) | 0.461 |  | 0.998 (0.987-1.009) | 0.698 |
| Platelet | 1.002 (1-1.004) | 0.025 |  | 1.002 (1-1.004) | 0.060 |  | 1.002 (0.998-1.006) | 0.270 |
| AST | 1.007 (0.998-1.016) | 0.141 |  | 1.003 (0.989-1.017) | 0.666 |  | 1.011 (1-1.023) | 0.057 |
| ALT | 1.003 (0.997-1.01) | 0.28 |  | 1.004 (0.995-1.013) | 0.353 |  | 1.005 (0.996-1.014) | 0.305 |
| Serum albumin | 0.948 (0.711-1.264) | 0.715 |  | 0.960 (0.698-1.32) | 0.802 |  | 1.011 (0.521-1.963) | 0.974 |
| Total bilirubin | 0.487 (0.268-0.888) | 0.019 |  | 0.368 (0.177-0.764) | 0.007 |  | 1.036 (0.334-3.217) | 0.951 |
| Total cholesterol | 0.999 (0.995-1.002) | 0.479 |  | 1.003 (0.998-1.007) | 0.252 |  | 0.989 (0.981-0.998) | 0.011 |
| Triglyceride | 1.001 (1-1.002) | 0.064 |  | 1.004 (1.001-1.006) | 0.002 |  | 1.001 (0.999-1.002) | 0.326 |
| HDL-cholesterol | 0.987 (0.976-0.999) | 0.031 |  | 0.989 (0.976-1.002) | 0.094 |  | 0.974 (0.947-1.002) | 0.073 |
| LDL-cholesterol | 0.998 (0.993-1.003) | 0.457 |  | 1.002 (0.996-1.008) | 0.554 |  | 0.994 (0.985-1.002) | 0.156 |
| Fasting blood glucose | 1 (0.995-1.005) | 0.984 |  | 0.999 (0.992-1.005) | 0.634 |  | 1.002 (0.994-1.01) | 0.568 |
| BARD | 1.171 (0.965-1.422) | 0.11 |  | 1.131 (0.875-1.462) | 0.347 |  | 1.213 (0.899-1.637) | 0.207 |
| NFS | 0.953 (0.858-1.059) | 0.375 |  | 0.968 (0.858-1.093) | 0.603 |  | 0.909 (0.733-1.128) | 0.386 |
| FIB-4 | 0.953 (0.785-1.157) | 0.628 |  | 0.952 (0.746-1.215) | 0.693 |  | 0.976 (0.703-1.354) | 0.884 |

Abbreviations: ER, estrogen receptor; PR, progesterone receptor; HER2, Human epidermal growth factor receptor 2; FSH, follicle stimulating hormone ; AST, aspartate aminotransferase; ALT, alanine aminotransferase; HDL, high-density lipoprotein; LDL, low-density lipoprotein; NFS, nonalcoholic fatty liver disease fibrosis score; FIB-4, fibrosis-4
