# Supplementary material for: Risk of fatty liver after long-term use of tamoxifen in patients with breast cancer
Source: PLoS One. 2020 Jul 30;15(7):e0236506. doi: 10.1371/journal.pone.0236506 (PMC7392315; doi:10.1371/journal.pone.0236506)
Supplement: S4 Table — (DOCX) [file pone.0236506.s007.docx]

**Supplementary Table 4. Univariable proportional hazards regression for death**

| **Variable** | **In total (N=911)** | |
| --- | --- | --- |
|  | **HR (95% CI)** | **p-value** |
| Fatty liver progression |  |  |
| No | 1 (Reference) |  |
| Yes | 0.875 (0.444-1.726) | 0.700 |
| Age (year) | 1.039 (1.011-1.068) | 0.006 |
| BMI (㎏/㎡) | 0.951 (0.866-1.044) | 0.293 |
| Diabetes | 3.020 (1.391-6.555) | 0.005 |
| Hypertension | 1.213 (0.577-2.55) | 0.610 |
| Cancer stage |  |  |
| ≤1 | 1 (Reference) |  |
| 2 | 1.858 (0.901-3.830) | 0.093 |
| ≥3 | 4.208 (1.772-9.992) | 0.001 |
| Pathology |  |  |
| Invasive ductal carcinoma | 1 (Reference) |  |
| Ductal carcinoma in situ | 0.306 (0.042-2.228) | 0.242 |
| Mucinous carcinoma | 0 (0-Inf) | 0.997 |
| Infiltrating lobular carcinoma | 0.829 (0.114-6.041) | 0.853 |
| Intraductal papilloma | 1.352 (0.185-9.862) | 0.766 |
| Tubular carcinoma | 0 (0-Inf) | 0.999 |
| Apocrine carcinoma | 0 (0-Inf) | 0.999 |
| Squamous carcinoma | 0 (0-Inf) | 0.999 |
| Medullary carcinoma | 0 (0-Inf) | 0.999 |
| Others | 0 (0-Inf) | 0.999 |
| Lymph node metastasis | 1.880 (1.008-3.508) | 0.047 |
| ER (Intermediate or High) | 0.303 (0.159-0.575) | <0.001 |
| PR (Intermediate or High) | 0.293 (0.149-0.578) | <0.001 |
| HER2 (Intermediate or High) | 0.464 (0.213-1.008) | 0.052 |
| p53 | 1.946 (1.015-3.734) | 0.045 |
| Ki67 (≥ 40%) | 1.334 (0.588-3.025) | 0.491 |
| Chemotherapy | 2.686 (0.955-7.55) | 0.061 |
| Radiotherapy | 1.489 (0.789-2.81) | 0.219 |
| Treatment duration (month) | 0.948 (0.927-0.968) | <0.001 |
| FSH | 1.001 (0.989-1.013) | 0.849 |
| Platelet | 0.995 (0.991-1) | 0.033 |
| AST | 1.006 (0.988-1.024) | 0.495 |
| ALT | 0.978 (0.95-1.006) | 0.124 |
| Serum albumin | 0.708 (0.359-1.395) | 0.319 |
| Total bilirubin | 1.559 (0.99-2.454) | 0.055 |
| Total cholesterol | 0.994 (0.985-1.003) | 0.166 |
| Triglyceride | 1 (0.996-1.004) | 0.946 |
| HDL-cholesterol | 0.943 (0.911-0.975) | 0.001 |
| LDL-cholesterol | 1.004 (0.992-1.016) | 0.513 |
| Fasting blood glucose | 0.997 (0.985-1.009) | 0.662 |
| BARD | 1.699 (1.086-2.66) | 0.020 |
| NFS | 1.404 (1.148-1.718) | 0.001 |
| FIB-4 | 1.301 (1.202-1.409) | <0.001 |
