# Supplementary material for: Risk of fatty liver after long-term use of tamoxifen in patients with breast cancer
Source: PLoS One. 2020 Jul 30;15(7):e0236506. doi: 10.1371/journal.pone.0236506 (PMC7392315; doi:10.1371/journal.pone.0236506)
Supplement: S5 Table — (DOCX) [file pone.0236506.s008.docx]

**Supplementary Table 5. Propensity score matching analysis for the risk factors associated with fatty liver progression (caliper 0.3)**

| **Variable** | **Multivariable** | |  |
| --- | --- | --- | --- |
|  | **HR (95% CI)** | | **p-value** |
| **All (N=488)** |  | |  |
| Treatment modality |  | |  |
| Control | 1 (Reference) | |  |
| Tamoxifen | 1.400 (1.018-1.927) | | 0.039 |
| Body mass index (㎏/㎡) | 1.069 (1.031-1.108) | | <0.001 |
| PR (Intermediate or High) | 1.658 (1.204-2.284) | | 0.002 |
| **Fatty liver (-) at baseline (N=370)** |  |  |  |
| Treatment modality |  |  |  |
| Control | 1 (Reference) |  |  |
| Tamoxifen | 1.942 (1.240-3.040) |  | 0.004 |
| Body mass index (㎏/㎡) | 1.077 (1.022-1.135) |  | 0.006 |
| Triglyceride | 1.004 (1.001-1.007) |  | 0.006 |
| **Fatty liver (+) at baseline (N=118)** |  |  |  |
| Treatment modality |  |  |  |
| Control | 1 (Reference) |  |  |
| Tamoxifen | 2.720 (1.435-5.156) |  | 0.002 |
| Body mass index (㎏/㎡) | 1.061 (0.982-1.145) |  | 0.132 |
| HER2 (Intermediate + High) | 1.584 (0.917-2.737) | | 0.099 |
| Radiotherapy | 1.856 (1.072-3.214) | | 0.027 |
| Total cholesterol | 0.990 (0.982-0.998) | | 0.02 |

Abbreviations: HR, hazard ratio; CI, confidence interval
