# Supplementary material for: Risk of fatty liver after long-term use of tamoxifen in patients with breast cancer
Source: PLoS One. 2020 Jul 30;15(7):e0236506. doi: 10.1371/journal.pone.0236506 (PMC7392315; doi:10.1371/journal.pone.0236506)
Supplement: S6 Table — (DOCX) [file pone.0236506.s009.docx]

**Supplementary Table 6. Propensity score matching analysis for the risk factors associated with fatty liver progression (caliper 0.1)**

| **Variable** | **Multivariable** | |  |
| --- | --- | --- | --- |
|  | **HR (95% CI)** | | **p-value** |
| **All (N=408)** |  | |  |
| Treatment modality |  | |  |
| Control | 1 (Reference) | |  |
| Tamoxifen | 1.452 (1.021-2.065) | | 0.038 |
| Body mass index (㎏/㎡) | 1.041 (0.998-1.086) | | 0.065 |
| PR (Intermediate or High) | 1.572 (1.113-2.219) | | 0.01 |
| **Fatty liver (-) at baseline (N=311)** |  |  |  |
| Treatment modality |  |  |  |
| Control | 1 (Reference) |  |  |
| Tamoxifen | 2.167 (1.323-3.550) |  | 0.002 |
| Body mass index (㎏/㎡) | 1.060 (0.998-1.126) |  | 0.058 |
| Triglyceride | 1.004 (1.002-1.007) |  | 0.001 |
| **Fatty liver (+) at baseline (N=97)** |  |  |  |
| Treatment modality |  |  |  |
| Control | 1 (Reference) |  |  |
| Tamoxifen | 2.464 (1.139-5.332) |  | 0.022 |
| Body mass index (㎏/㎡) | 1.051 (0.959-1.151) |  | 0.288 |
| HER2 (Intermediate + High) | 1.697 (0.932-3.088) | | 0.083 |
| Radiotherapy | 1.140 (0.609-2.135) | | 0.682 |
| Total cholesterol | 0.990 (0.981-0.999) | | 0.029 |

Abbreviations: HR, hazard ratio; CI, confidence interval
